# Impact of physical activity on Chinese college students’ “going into the world” psychology: chain mediating effects of hardiness and positive academic emotions

**DOI:** 10.3389/fpsyg.2025.1711343

**Published:** 2025-12-05

**Authors:** Xiangxuan Guo, Dawei Cao, Donghuan Bai, Dong Wu, Lulu Kuang

**Affiliations:** 1 School of Physical Education, Huaibei Normal University, Huaibei, Anhui, China; 2 Chinese Wushu Academy, Beijing Sport University, Beijing, China

**Keywords:** physical activity, “going into the world” psychology, hardiness, positive academic emotions, chain mediation, college students

## Abstract

**Introduction:**

Based on positive psychology and emotion regulation theory, this study constructed a chain mediation model to explore the mechanism through which physical activity influences Chinese college students’ “Going into the World” psychology, specifically examining the mediating roles of hardiness and positive academic emotions.

**Methods:**

A total of 755 college students were surveyed using cluster random sampling. The instruments employed included the Physical Activity Rating Scale-3 (PARS-3), the Hardiness Scale (HS), the Academic Emotions Questionnaire (AEQ), and the Scale of Going into the World (SGIW). Data were analyzed using correlation analysis, regression analysis, and bootstrap mediation tests (Model 6, 5000 samples).

**Results:**

Correlation analysis revealed significant positive correlations between physical activity, hardiness, positive academic emotions, and “Going into the World” psychology. Regression analysis indicated that physical activity not only directly and positively predicted “Going into the World” psychology (B = 0.1489, *p* < 0.001) but also significantly predicted hardiness and positive academic emotions. Mediation analysis using the Bootstrap method confirmed significant indirect effects via three pathways: the independent mediating effect of hardiness (Effect = 0.0415, 95% CI [0.0249, 0.0621]), the independent mediating effect of positive academic emotions (Effect = 0.0086, 95% CI [0.0015, 0.0205]), and their chain mediating effect (Effect = 0.0039, 95% CI [0.0006, 0.0108]). The total mediating effect accounted for 26.61% of the overall effect, with hardiness exhibiting a more substantial mediating role.

**Discussion:**

The results indicate that physical activity enhances college students’ “Going into the World” psychology both directly and indirectly by improving their hardiness and positive academic emotions. This study provides a theoretical foundation and practical pathways for promoting college students’ mental health and social adaptation through physical activity interventions, highlighting the particular importance of fostering psychological hardiness.

## Introduction

“Going into the World” psychology refers to an individual’s affirmation of and engagement with social reality. It encompasses psychological processes, such as motivation activation, goal achievement, and need fulfillment, primarily manifested as the active pursuit of goals (Wang, 2011). In other words, it refers to an individual’s psychological tendency to actively engage with reality, proactively adapt to the social environment, and seek self-actualization. In the context of the new era, Chinese college students face increasingly intense academic competition and employment pressure. “Going into the World” psychology has become an important indicator for assessing their psychological health and social adaptability (Ye et al., 2020). Cultivating proactive social integration and self-actualization abilities among college students is a key focus of global higher education. Recent international research has emphasized the role of positive psychological emotions, personality, and goals in promoting social adaptation among college students (Koolaee and Malekitabar, 2025; Lin and Jiang, 2023). In 2020, Chinese state leaders emphasized at a symposium for scientists that “we should attach importance to the cultivation of young talents and enhance their sense of social responsibility and innovative spirit,” highlighting the significance of college students actively integrating into society and realizing their self-worth (Xi, 2020). The “Guidelines for Psychology Health Education of College Students” issued by the Ministry of Education of China clearly states that efforts should be made to enhance the social adaptability of college students and promote their overall healthy development (Ministry of Education of the People’s Republic of China, 2018).

Physical activity, a crucial means of enhancing physical and mental health, has been widely confirmed to positively influence individuals’ psychological state and social adaptability (Cai et al., 2025). Extensive research indicates that regular participation in physical activity can significantly improve an individual’s psychological resilience, emotion regulation abilities, and willingness to engage in social participation (Zhang et al., 2025; Chu et al., 2023; Yang and Lu, 2025). However, current research on the relationship between physical activity and “Going into the World” psychology remains relatively scarce, particularly regarding its underlying mediating mechanisms. Although some studies suggest that physical activity may promote social adaptive behaviors by enhancing self-efficacy (Yu et al., 2025) and perceived social support (He and Wang, 2025), these variables often fail to fully reveal the intrinsic psychological processes.

“Hardiness,” a positive psychological resource, refers to an individual’s tendency to exhibit commitment, control, and a sense of challenge when facing pressure and adversity. It has been confirmed as an important factor in coping with adversity and promoting psychological adaptation (Joana et al., 2021). Additionally, “positive academic emotions,” such as enjoyment, hope, and a sense of achievement, are considered an important affective foundation for academic success and social adaptation (Zhang et al., 2022). According to the broaden-and-build theory of positive emotions (Fredrickson, 2001), positive emotions can broaden individuals’ momentary thought–action repertoires and build their enduring personal resources, which may include enhanced social connectedness and goal-directed behaviors. Research shows that physical activity can enhance an individual’s psychological resilience (Niu et al., 2025), thereby promoting positive emotional experiences (Thom et al., 2020). Both hardiness and positive academic emotions may collectively influence college students’ “Going into the World” psychology.

Based on this context, this study assumes that physical activity directly and positively predicts the development of “Going into the World” psychology among Chinese college students. In addition, by introducing “hardiness” and “positive academic emotions” as mediating variables, a chain mediation model is constructed to explore the mechanism through which physical activity influences the “Going into the World” psychology of Chinese college students. This study hypothesizes that physical activity can directly predict “Going into the World” psychology and indirectly enhance college students’ social adaptability by improving “hardiness” and “positive academic emotions.” The findings of this research aim to deepen the understanding of the psychological benefits of physical activity and provide a theoretical basis and practical pathways for universities to promote college students’ social adaptability through physical activity interventions.

## Hypothetical chain mediation model of the relationship between physical activity and “going into the world” psychology

### The predictive effect of college students’ physical activity on “going into the world” psychology

In recent years, with increasing social competition and pressure, the cultivation of college students’ “Going into the World” psychology has become an important objective of higher education and psychology education. Physical activity, as a form of active lifestyle, has been confirmed to enhance individuals’ psychological adaptability and social behavioral performance. For example, physical activity can enhance college students’ social adaptability by improving their self-efficacy and sense of social support (Qiao and Xiao, 2025). Yuan Gao et al., also noted that college students who regularly participate in physical activity demonstrate higher levels of career decision-making self-efficacy and social initiative (Gao et al., 2023). Therefore, this study proposes Hypothesis 1 (H1):

*H*1: Physical activity has a positive predictive effect on college students' “Going into the World” psychology.

### The mediating effect of hardiness

Hardiness refers to the psychological trait characterized by commitment, control, and challenge when an individual faces pressure and adversity. It is an important resource for coping with adversity and promoting psychological adaptation. Research shows that physical activity can significantly enhance an individual’s psychological resilience. For instance, Haixia Peng et al., found that college students who engage in long-term physical activity have a significantly higher level of hardiness than those who do not exercise (Peng et al., 2017). Physical activity promotes the internalization and accumulation of resilience resources by providing challenging situations and successful experiences (Hwan, 2020). Individuals with a high level of hardiness are more likely to actively cope with challenges in their social environment, demonstrating a stronger willingness and ability to adapt socially. Therefore, hardiness may play a mediating role in the relationship between physical activity and “Going into the World” psychology. Based on this, Hypothesis 2 (H2) is proposed:

*H*2: Hardiness mediates the relationship between college students' physical activity and “Going into the World” psychology.

### The mediating effect of positive academic emotions

Positive academic emotions refer to positive emotional states, such as enjoyment, hope, and pride, experienced by individuals during the academic process. Research has found that physical activity can effectively enhance an individual’s emotion regulation abilities and positive emotional experiences. For example, research by Ning Dong et al., showed that physical activity significantly improves college students’ positive emotions by promoting endorphin release and emotional venting (Dong et al., 2025). Positive academic emotions not only enhance learning engagement and academic achievement but also strengthen an individual’s identification with and willingness to adapt to social roles. For instance, Haihong Li et al., found that positive emotional experiences significantly predict college students’ motivation for career exploration and social participation (Li et al., 2021). Therefore, positive academic emotions may play a positive mediating role in the relationship between physical activity and “Going into the World” psychology. Based on this, Hypothesis 3 (H3) is proposed:

*H*3: Positive academic emotions play a positive mediating role in the relationship between physical activity and “Going into the World” psychology.

### The chain mediating role of hardiness and positive academic emotions

Physical activity may influence “Going into the World” psychology not only through the independent mediating effects of hardiness or positive academic emotions but also through a chain pathway involving both, exerting a synergistic effect. According to positive psychology and emotion regulation theory, individuals with a high level of hardiness are more likely to adopt positive cognitive strategies to cope with stress, thereby maintaining a higher level of positive emotions. Positive emotions can further broaden an individual’s cognitive–behavioral resources, promoting the development of socially adaptive behaviors. For example, research by Baoxia Zhou et al., showed that psychological resilience enhances college students’ social adaptability indirectly by boosting positive emotions (Zhou and Liu, 2025). In the context of physical activity, individuals accumulate resilience resources through physical challenges experiences of achievement. This, in turn, stimulates positive emotional experiences, ultimately enhancing their tendency toward “Going into the World” psychology. Based on this, Hypothesis 4 (H4) is proposed:

*H*4: Hardiness and positive academic emotions play a chain mediating role in the relationship between physical activity and “Going into the World” psychology.

**Figure 1:**
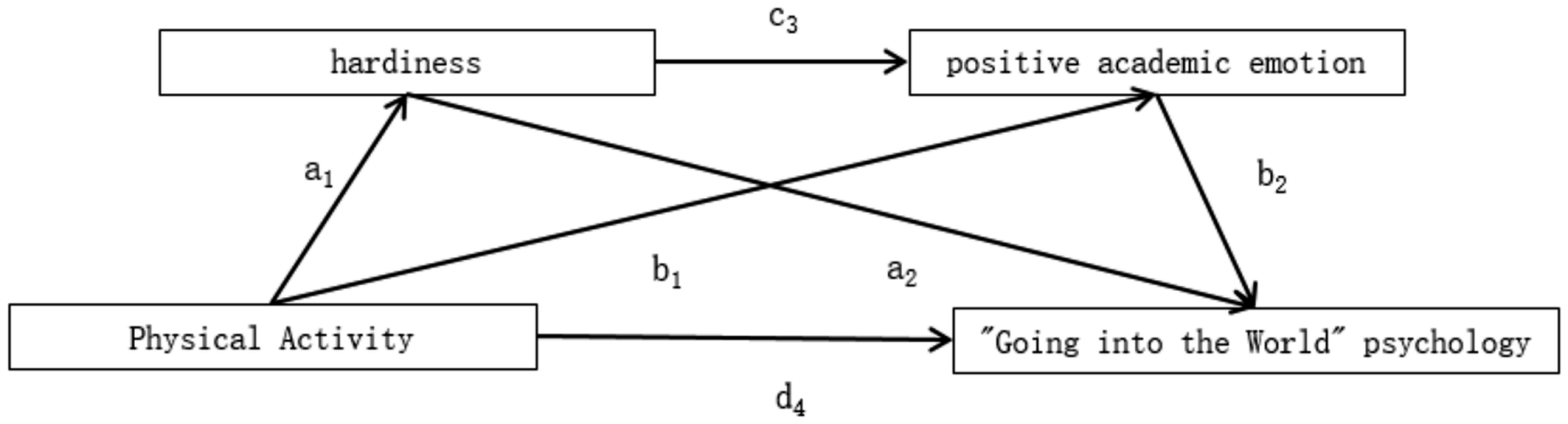
Hypothetical chain mediation model of the influence of physical activity on “going into the world” psychology.

In summary, this study constructed the chain mediation model shown in Figure 1 to reveal the internal mechanism through which physical activity influences college students’ “Going into the World” psychology.

## Research methods

### Participants and testing procedure

This study used the cluster random sampling method. Based on the convenience of the author’s investigation, college students from three universities in Beijing and Anhui Province, China, were selected as survey participants. The sample included students from 12 different majors, spanning from freshman to senior year. A total of 16 classes were randomly selected using a random number generator. The sample size was determined using the “Monte Carlo Power Analysis for Indirect Effects” tool (website: https://schoemanna.shinyapps.io/mc_power_med/). The parameters for the Monte Carlo power analysis for indirect effects were set as follows: Model: Two Serial Mediators; Target Power: 0.8; Sample Size Steps: 1; Number of Replications: 1000; Monte Carlo Draws per Replication: 5000; Random Seed: 1234; Confidence Level: 95%. The required correlation coefficients and standard deviations were obtained from Table 1 for the analysis. The estimated sample size was 735. Our final sample size of 755 exceeded this requirement.

**Table 1:** Means, standard deviations, and correlation results of variables.

Variable	M	SD	PE	H	PAE	GIWP
PE	31.8106	22.17491	1			
H	68.8768	11.27528	0.423**	1		
PAE	67.8517	10.31798	0.399**	0.477**	1	
GIWP	51.6675	8.29668	0.625**	0.595**	0.434**	1

*p < 0.05, **p < 0.01, ***p < 0.001; PE, Physical activity; H, Hardiness; PAE, Positive Academic Emotions; GIWP, “Going into the World” psychology.

To ensure the participants’ right to informed consent and adherence to the principle of voluntary participation, they were informed of the confidentiality measures regarding the survey content and the purpose of the data collection before the survey commenced. Counselors or course instructors assisted with the administration of the survey. Questionnaires were distributed by counselors or course instructors via class groups. A total of 788 questionnaires were collected. Based on the screening criteria, such as completeness of information, presence of patterned responses, and omissions, each questionnaire was individually reviewed. After excluding invalid questionnaires, 755 valid questionnaires were retained, yielding an effective response rate of 95.81%. Among the participants, 343 were male (45.43%) and 412 were female (54.57%) individuals; 194 were freshmen (25.7%), 174 were sophomores (23.05%), 181 were juniors (23.97%), and 206 were seniors (27.28%). A total of 367 participants were from urban areas (48.61%) and 388 were from rural areas (51.39%). The participants’ majors included the following: Philosophy (50, 6.62%), Economics (68, 9%), Law (73, 9.67%), Education (95, 12.58%), Literature (51, 6.76%), History (66, 8.74%), Science (82, 10.86%), Engineering (52, 6.89%), Agriculture (53, 7.02%), Management (69, 9.14%), Art (59, 7.82%), and Interdisciplinary Studies (37, 4.9%).

### Research tools

#### Physical activity rating scale-3

The Physical Activity Rating Scale-3 (PARS-3), originally developed by a Japanese scholar, Hashimoto Kimio, and revised by Deqing Liang et al., was used. This questionnaire reflects participants’ physical activity levels and is a widely used tool in China for measuring physical activity volume. The scale uses a 5-point Likert scoring system (1–5 points) and consists of three dimensions: exercise intensity (A), duration per session (B), and frequency of exercise (C). The physical activity score is calculated as A*(B-1)*C. This formula is designed to yield an overall index of physical activity volume, integrating intensity, duration, and frequency into a single metric. The total score ranges from 0 to 100, with scores ≤19 indicating low physical activity, 20–42 indicating moderate physical activity, and ≥43 indicating high physical activity. A higher score indicates a greater volume of physical activity. The retest reliability of the scale was 0.82, and Cronbach’s *α* coefficient was 0.74. The scale demonstrated good internal consistency and high reliability (Liang, 1994).

#### College student hardiness scale

The College Student Hardiness Scale, developed by Guohua Lu and Baoyong Liang in 2008, was used. Based on foreign theories of hardiness, this scale includes four dimensions: tenacity, commitment, control, and challenge, comprising 27 items. It uses a 4-point Likert scoring method: “Completely consistent” (4 points), “Consistent” (3 points), “Somewhat consistent” (2 points), and “Completely inconsistent“(1 point). The factor loadings of all items were above 0.40, meeting the conventional threshold for adequate item representation (Lu and Liang, 2008). The internal consistency (α coefficients) values for the control, commitment, challenge, and tenacity subscales were 0.785, 0.747, 0.784, and 0.802, respectively, and 0.910 for the full scale. The retest reliability for control, commitment, challenge, tenacity, and the full scale was 0.91, 0.89, 0.92, 0.91, and 0.92, respectively, with all *p*-values less than 0.01. In terms of construct validity, the model explained 46.45% of the total variance, with an RMSEA of 0.059 and NFI, NNFI, and CFI values all above 0.9, further indicating the stability of the factor model (Lu and Liang, 2008).

#### College student academic emotion questionnaire

The concept of academic emotions was proposed by Pekrun et al. (2002). It refers to various emotional experiences related to students’ academics during teaching or learning processes, including happiness, boredom, disappointment, anxiety, and anger (Yu and Dong, 2005). In 2009, Xiancai Xu developed the “College Student Academic Emotion Scale” based on the characteristics of Chinese college students (Xu and Gong, 2011). The questionnaire consists of 50 items, which are divided into four sub-questionnaires: positive activity-focused academic emotions, positive outcome-focused academic emotions, negative activity-focused academic emotions, and negative outcome-focused academic emotions. This study used the positive academic emotion sub-questionnaire from the “College Student Academic Emotion Scale,” which includes two subscales—positive activity-focused and positive outcome-focused academic emotions—totaling 22 items. Positive activity-focused academic emotions include the dimensions of happiness, relaxation, and autonomy, comprising 15 items; positive outcome-focused academic emotions include the dimension of sense of achievement, comprising seven items. The scale uses a 5-point Likert scoring method, ranging from 1 (Very inconsistent) to 5 (Very consistent), with a total score range of 22–110. A higher score indicates stronger experiences of these emotions. The Cronbach’s *α* coefficient for the full scale was 0.898. In this study, the Cronbach’s α coefficient for the positive academic emotion sub-questionnaire was 0.920 (Min et al., 2025). The Cronbach’s α coefficients for the two subscales were 0.84 and 0.84, and the split-half reliabilities were 0.84 and 0.80, respectively. The fit indices of the confirmatory factor analysis model for the questionnaire all met acceptable standards (Xu and Gong, 2011).

#### Scale of going into the world

The Scale of Going into the World (SGIW) from the “Scale of Going into the World and Leaving the world” (SGIW&LW) was used. The first-order factor structure of the SWIS&LW (Factor 1: striving spirit, Factor 2: concern for results, Factor 3: ordinary mind, and Factor 4: low demands) yielded the following results: χ^2^(131, N = 361) = 263.64 (*p* = 0.00), RMSEA = 0.09, CFI = 0.92, ECVI = 0.95, NFI = 0.81, IFI = 0.89, NNFI = 0.88, CFI = 0.89, and RMR = 0.067, indicating an acceptable model. The second-order factors consist of two factors: “Going into the World” and “Leaving the world.” The Cronbach’s α coefficients, split-half reliabilities, and retest reliabilities for the first-order and second-order factors of the scale ranged between 0.60 and 0.76. The correlations between the first-order and second-order factors of the scale and the criterion scales (the Life Satisfaction Questionnaire, the Rosenberg Self-Esteem Scale, the Self-Description Questionnaire, and the Big Five Personality Scale) ranged from −0.28 to 0.29. The sum of the scores for striving spirit and concern for results constitutes the total score for the “Going into the World” dimension, reflecting the respondent’s active pursuit of goals. A 7-point Likert scoring method was used, with a minimum score of 1 and a maximum score of 7: “Strongly disagree” (1 point), “Disagree” (2 points), “Somewhat disagree” (3 points), “Neutral” (4 points), “Somewhat agree” (5 points), “Agree” (6 points), and “Strongly agree” (7 points; Wang, 2011). This scale has been primarily validated and applied within the Chinese cultural context (Wang, 2011). However, its constructs share conceptual similarities with Western concepts of proactive personality and approach motivation, suggesting potential for cross-cultural relevance, although further validation in international samples is warranted.

### Data processing

Valid data were imported into SPSS 26.0 statistical analysis software. Pearson correlation analysis, regression analysis, and chain mediation analysis were performed. The chain mediation analysis utilized the multiple mediation effect analysis method proposed by Hayes, using the Process plugin (Version 5.0) of SPSS for mediation effect testing. The model was specified as Model 6, with X = physical activity, M1 = hardiness, M2 = positive academic emotions, and Y = “Going into the World” psychology. Bootstrap Samples were set to 5,000, with the confidence interval set at 95%. If the 95% confidence interval did not include 0, it indicated statistical significance (Hayes, 2013). Descriptive statistics (means and standard deviations) for all key variables are presented in Table 1. In all regression and mediation analyses, student data such as sex, academic year, and majors were included as covariates to control for their potential confounding effects.

The mediation effects in the multiple mediation model included independent mediation effects and chain mediation effects (Kong and Wang, 2022). The independent mediation effects were “physical activity→hardiness→‘going into the world’ psychology” and “physical activity→positive academic emotions→‘going into the world’ psychology.” The chain mediation effect was “physical activity→hardiness→positive academic emotions→‘going into the world’ psychology.” The total mediation effect was the sum of these two mediation effects.

## Results

### Common method bias test

As all variables in this study were measured using questionnaires, common method bias was a potential concern. Therefore, Harman’s single factor test (Podsakoff et al., 2003) was conducted by performing an unrotated exploratory factor analysis on all variables. The results showed that there were three factors with eigenvalues greater than 1. The variance explained by the first factor was 34.535%, which is below the critical threshold of 40%, indicating no significant common method bias.

### Descriptive statistics and correlation analysis of physical activity, hardiness, positive academic emotions, and “going into the world” psychology

Correlation analysis showed that physical activity was significantly positively correlated with “Going into the World” psychology (*r* = 0.625, *p* < 0.01), hardiness (*r* = 0.423, *p* < 0.01), and positive academic emotions (*r* = 0.399, *p* < 0.01). “Going into the World” psychology was significantly positively correlated with hardiness (*r* = 0.595, *p* < 0.01) and positive academic emotions (*r* = 0.434, *p* < 0.01). Hardiness was significantly positively correlated with positive academic emotions (*r* = 0.477, *p* < 0.01). When the level of physical activity increased, the “Going into the World” psychology also increased. When an individual’s hardiness or positive academic emotions improved, their “Going into the World” psychology also improved accordingly (Table 1).

### Testing the chain mediation model of physical activity affecting “going into the world” psychology: the roles of hardiness and positive academic emotions

To explore the predictive relationships between physical activity, hardiness, positive academic emotions, and “Going into the World” psychology, regression analysis was conducted based on correlation analysis, with the first three variables treated as independent variables and “Going into the World” psychology treated as the dependent variable. The specific statistical results are shown in Table 2.

**Table 2:** Results of regression analysis examining the mediating effects in the relationship between physical activity and college students’ “going into the world” psychology (*n* = 755).

Variable	Model 1: H		Model 2: PAE		Model 3: GIWP	
	Coefficient	Std. error	Coefficient	Std. error	Coefficient	Std. error
PE	0.1649***	0.0176	0.1178***	0.0168	0.1489***	0.0111
H			0.323***	0.331	0.2515***	0.0224
PAE					0.0729**	0.0233
Sex	0.3125	0.7117	−0.22	0.6457	1.3006**	0.4115
Origin	−0.6966	0.7063	0.1351	0.6412	−0.6564	0.4085
Major	0.0105	0.0974	−0.2214*	0.0884	0.2442***	0.0565
Grade	3.1443***	0.3447	0.3332	0.3296	0.7648***	0.2102
Constant	45.6605	98.0968	264.0002	89.0059	−224.1649***	57.0419
R^2^	0.2717		0.2852		0.5518	
△*R*^2^	0.2668		0.2794		0.5476	
*F*-value	55.8795		49.7316		131.3765	

*p < 0.05, **p < 0.01, ***p < 0.001; PE, Physical activity; H, Hardiness; PAE, Positive Academic Emotions; GIWP, “Going into the World” psychology.

As shown in Table 2, in Model 1, the independent variable included in the regression equation was physical activity, which had a significant positive effect on hardiness (B = 0.1649, *p* < 0.001). This suggests that for every one-unit increase in physical activity, hardiness increased by 0.1649 units, indicating a positive predictive effect. Model 2 used positive academic emotions as the dependent variable to examine the effects of physical activity and hardiness on positive academic emotions. It was found that physical activity had a significant positive predictive effect on positive academic emotions (B = 0.1178, *p* < 0.001), meaning that for every one-unit increase in physical activity, the positive academic emotion score increased by 0.1178 units. Hardiness also had a significant positive predictive effect on positive academic emotions (B = 0.323, *p* < 0.001), indicating that for every one-unit increase in hardiness, the positive academic emotion score increased by 0.323 units.

Model 3 is the primary focus of this study. It used “Going into the World” psychology as the dependent variable to examine the effects of physical activity, hardiness, and positive academic emotions on college students’ “Going into the World” psychology. Among the control variables, sex, majors, and grade had significant effects on college students’ “Going into the World” psychology. According to Model 3, after including the two mediating variables, hardiness and positive academic emotions, the direct predictive effect of physical activity on college students’ “Going into the World” psychology remained significant (B = 0.1489, *p* < 0.001). This means that, after controlling for the mediating variables and relevant covariates, for every one-unit increase in physical activity, college students’ “Going into the World” psychology variable score increased by 0.1489. Research Hypothesis 1 was supported. Furthermore, the positive effects of hardiness (B = 0.2515, *p* < 0.001) and positive academic emotions (B = 0.0729, *p* < 0.01) on college students’ “Going into the World” psychology were also significant. For every one-unit increase in hardiness, college students’ “Going into the World” psychology variable score increased by 0.2515; for every one-unit increase in positive academic emotions, the score increased by 0.0729.

As shown in Table 2, physical activity significantly predicted hardiness (Model 1), accounting for 27.17% of its variance (R^2^ = 0.2717). When predicting positive academic emotions (Model 2), the combined effect of physical activity and hardiness explained 28.52% of the variance (R^2^ = 0.2852). The full model (Model 3), including physical activity, hardiness, and positive academic emotions, explained 55.18% of the variance in ‘Going into the world’ psychology (R^2^ = 0.5518).

**Figure 2:**
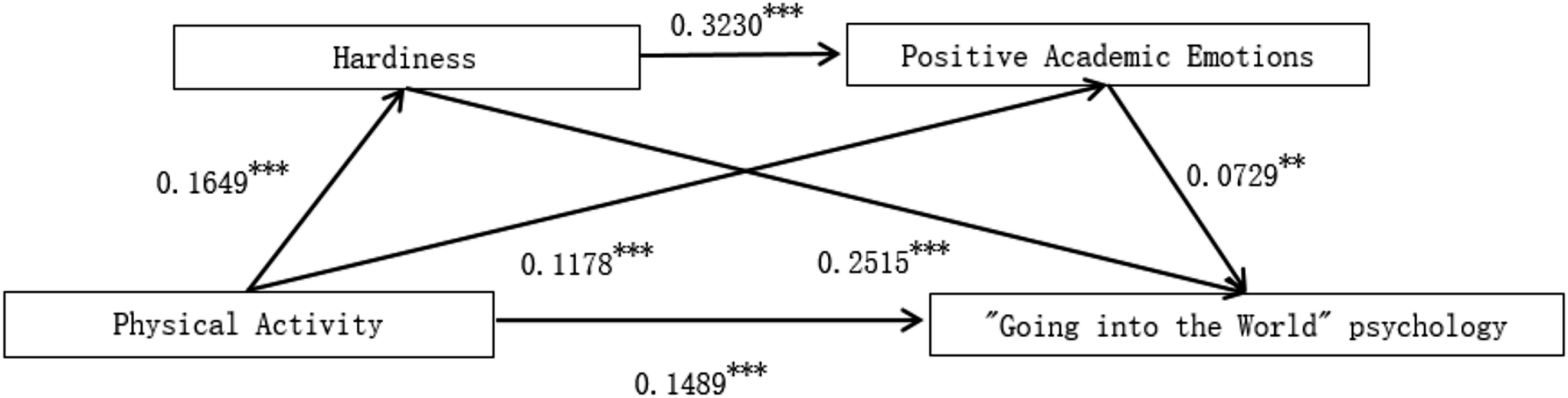
Path coefficients of the multiple mediation regression model examining the relationship between physical activity and college students’ “going into the world” psychology.

In summary, physical activity positively predicts college students’ hardiness, indicating that physical activity promotes the cultivation and development of hardiness. Physical activity and hardiness positively predict college students’ positive academic emotions, indicating that they can enhance positive academic emotions. Physical activity, hardiness, and positive academic emotions positively predict college students’ “Going into the World” psychology. Higher levels of physical activity are associated with higher levels of hardiness and positive academic emotions, leading to higher scores on “Going into the World” psychology. The path coefficients of the multiple mediation regression model are shown in Figure 2.

This study employed the bias-corrected nonparametric percentile bootstrap method, with 5,000 repeated samples, to calculate 95% confidence intervals for testing the multiple mediation effects. According to Table 3, the direct effect of physical activity on college students’ “Going into the World” psychology, the independent mediating effects of hardiness and positive academic emotions, and the chain mediating effect of hardiness and positive academic emotions all had bootstrap 95% confidence intervals whose upper and lower limits did not include zero. This indicates that hardiness and positive academic emotions play significant mediating roles in the relationship between physical activity and college students’ “Going into the World” psychology. In other words, physical activity can not only directly affect college students’ “Going into the World” psychology but also influence it through the independent mediating effects of hardiness and positive academic emotions, as well as their chain mediating effect. Research Hypotheses 2 and 3 were supported.

**Table 3:** Bootstrap analysis of the significance and effect sizes of mediating effects.

Influence pathway	Effect	Boot SE	Boot 95% CI lower	Boot 95% CI upper	Relative percentage
PE → H → GIWP (Ind1)	0.0415	0.0093	0.0249	0.0621	20.45%
PE → PAE → GIWP (Ind2)	0.0086	0.0055	0.0015	0.0205	4.24%
PE → H → PAE → GIWP (Ind3)	0.0039	0.0029	0.0006	0.0108	1.92%
Total indirect effect	0.054	0.0101	0.035	0.0746	26.61%
Direct effect	0.1489	0.0111	0.1272	0.1706	73.39%
Total effect	0.2029	0.0113	0.1807	0.225	—

PE, Physical activity; H, Hardiness; PAE, Positive Academic Emotions; GIWP, “Going into the World” psychology.

Furthermore, as shown in Table 3, the direct effect of physical activity on “Going into the World” psychology was 0.1489, the mediating effect of hardiness was 0.0415, the mediating effect of positive academic emotions was 0.0086, and the chain mediating effect of hardiness and positive academic emotions was 0.0039. The total mediating effect of these three pathways was 0.054, accounting for 26.61% of the total effect. Compared to the mediating effect of positive academic emotions, the mediating effect of hardiness was larger.

## Discussion

Based on positive psychology and emotion regulation theory, this study constructed and validated a chain mediation model to systematically explore the internal mechanism through which physical activity influences the “Going into the World” psychology of Chinese college students. The results supported all hypotheses, indicating that physical activity can directly enhance college students’ “Going into the World” psychology and exert an indirect effect through the independent mediating roles of hardiness and positive academic emotions, as well as their chain mediation pathway.

First, this study found that physical activity has a significant positive predictive effect on college students’ “Going into the World” psychology (H1 supported). This result is consistent with previous research, confirming that physical activity, as a proactive lifestyle, is an effective way to cultivate college students’ “Going into the World” psychology (Qin et al., 2023; Hong, 2014; Tang et al., 2025). The sense of achievement, awareness of rules, and teamwork experience gained through physical activity may be directly transferred to how individuals cope with social challenges, thereby encouraging them to integrate into and adapt to society more actively.

Second, the mediation effect analysis showed that hardiness plays an important mediating role in the relationship between physical activity and “Going into the World” psychology (H2 supported). This finding validates psychological resilience theory. Physical activity, effectively promotes the internalization and enhancement of resilience traits such as commitment, control, and challenge tendency by providing continuous physical challenges and successful experiences of overcoming difficulties (Guo et al., 2021). Individuals with high hardiness are more likely to perceive pressures in the social environment as manageable challenges rather than threats, thereby demonstrating stronger motivation for goal pursuit and social adaptive behaviors (Shang and Yang, 2021). This may explain why the mediating effect of hardiness (20.45%) was substantially larger than that of positive academic emotions (4.24%). In the context of intense academic and employment challenges faced by Chinese college students, a resilient personality trait may be more critical than transient positive emotions in sustaining long-term social adaptive behaviors.

Third, it was found that positive academic emotions also play a significant mediating role between the two (H3 supported). This indicates that physical activity not only affects individuals’ cognitive traits (hardiness) but also exerts influence through emotional pathways. Regular physical activity can effectively promote positive emotional experiences, such as pleasure, hope, and a sense of achievement. According to the broaden-and-build theory of positive emotions (Fredrickson et al., 2000), these positive academic emotions, in turn, broaden students’ cognitive–behavioral resources, enabling them to approach academic and social tasks with a more optimistic, creative, and energetic mindset, thereby strengthening their willingness to integrate into society and achieve goals (Zhang et al., 2022).

Most importantly, this study revealed a statistically significant chain mediation pathway of “hardiness → positive academic emotions” (H4 supported). This suggests that the promoting effect of physical activity on “Going into the World” psychology involves a sequential internal process: physical activity first consolidates the foundation of students’ hardiness, and this positive psychological trait, in turn, enables them to better employ positive cognitive reappraisal strategies to cope with academic stress, thereby maintaining and experiencing higher levels of positive emotions (Zhou and Liu, 2025). This synergistic effect of “resilience” and “positive emotions” is ultimately more effectively translated into “social adaptation” behavior. This chain pathway aligns with the classic psychological model, wherein stable traits (hardiness) influence emotional states (positive academic emotions), which, in turn, foster behavioral tendencies (‘Going into the World’ psychology). It also resonates with the broaden-and-build theory (Fredrickson et al., 2000), which posits that positive emotions broaden individuals’ thought–action repertoires, thereby promoting adaptive behaviors.

The direct effect, even after accounting for the mediators, suggests that our model, while robust, does not capture the full picture. Other unmeasured mechanisms, such as the neurobiological benefits or improved social skills mentioned earlier, likely contribute directly to the outcome. Among the indirect pathways, the independent mediating effect of hardiness (20.45%) was substantially larger than that of positive academic emotions (4.24%) and the chain mediation effect (1.92%). This clearly indicates that cultivating resilient psychological qualities is a more central mechanism in this process. It is important to acknowledge that the variance explained by the serial mediation pathway, while statistically significant, was notably modest (1.92%). Therefore, we consider this a preliminary yet theoretically meaningful finding. It provides initial evidence for a specific sequence of psychological processes, but its practical contribution in this context is limited and warrants further verification in future studies.

## Conclusion

In summary, this study demonstrates that physical activity not only directly enhances Chinese college students’ “Going into the World” psychology but also exerts its influence through three significant indirect pathways: the independent mediation of hardiness, the independent mediation of positive academic emotions, and their sequential chain mediation. Among these, the mediating role of hardiness emerges as the most substantial mechanism.

These findings offer clear, practical implications for higher education practitioners. First, universities should strategically recognize and promote physical activity beyond its physical health benefits, framing it as a valuable intervention for cultivating essential psychological resources and enhancing social adaptability. Second, structured physical activity programs—such as mandatory fitness courses, diverse sports clubs, and intramural leagues—should be systematically designed and widely accessible to encourage consistent student participation. By doing so, educators can proactively cultivate students’ hardiness and positive academic emotions, thereby ultimately empowering them to actively integrate into society and confidently pursue self-realization. Future interventions should focus on developing activity protocols that are specifically tailored to enhance these psychological traits.

## Data Availability

The original contributions presented in the study are included in the article/supplementary material, further inquiries can be directed to the corresponding authors.
